# The Swedish translation and cultural adaptation of the Measure of Moral Distress for Healthcare Professionals (MMD-HP)

**DOI:** 10.1186/s12910-021-00722-3

**Published:** 2021-11-12

**Authors:** Catarina Fischer-Grönlund, Margareta Brännström

**Affiliations:** Department of Nursing, Umeå University, Linnaeus v 9, 90736 Umeå, Sweden

**Keywords:** Measure moral distress, Health care professionals, Questionnaire, Swedish translation

## Abstract

**Background:**

Moral distress has been described as an emotionally draining condition caused by being prevented from providing care according to one’s convictions. Studies have described the impact of moral distress on healthcare professionals, their situations and experiences. The Measure of Moral Distress for Healthcare Professionals (MMD-HP) is a questionnaire that measures moral distress experienced by healthcare professionals at three levels: patient, system and team. The aim of this project was to translate and make a cultural adaption of the MMD -HP to the Swedish context.

**Methods:**

The questionnaire comprises 27 items, rated according to frequency and intensity on a five-point Likert scale (0–4). The procedure for translating MMD-HP followed WHO guidelines (2020). These entailed a forward translation from English to Swedish, a back translation, expert panel validation, pretesting and cognitive face-to-face interviews with 10 healthcare professionals from various professions and healthcare contexts.

**Results:**

The Swedish version of MMD-HP corresponds essentially to the concept of the original version. Parts of some items’ had to be adjusted or removed in order to make the item relevant and comprehensible in a Swedish context. Overall, the cognitive interviewees recognized the content of the items which generally seemed relevant and comprehensible.

**Conclusion:**

The Swedish version of MMD-HP could be a useful tool for measuring moral distress among healthcare professionals in a Swedish healthcare context.

## Background

Moral distress was described by Jameton [1] as a condition with painful feelings of frustration, anger and anxiety caused by being prevented from providing care in accordance with one’s moral convictions. In contrast to moral uncertainty, which means being unsure about what is the right action, moral distress arises when a person makes a moral judgement about the right course to take but is prevented from acting on it [2]. Rushton et al. [3] describes moral distress as an emotional response to distressing situations, involving value conflicts and impacting on empathy, experience of memory, cognitive attunement and moral sensitivity. Moral distress has been described as a subjective experience, based on one’s personal value system and a compromised moral integrity [4]. It is an emotionally draining condition caused by being obliged to act in ways which conflict with one’s values [5], leading to a risk of burn-out [6] and a reason for leaving the profession [7]. Hence, being constantly prevented from acting in accordance with one’s professional standards regarding best treatment and care entails a risk of compromising one’s long-term core values, and both professional and personal integrity [8, 9].

Moral distress among healthcare professionals has been studied in various healthcare contexts including oncology [10], palliative care [3], psychiatry [11], pediatric care [12], surgical and medical care [13], emergency [14] and intensive care [15–17]. In a study Hancock et al. [16] revealed three principal issues underlying the development of moral distress; namely, organizational issues, exposure to highly intense situations and poor team experiences. Several studies found that organizational constraints reduced the standard of patient care [18] and low staffing levels were found to be important sources of moral distress [11, 16, 19, 20].

RNs have reported feeling moral distress from having to deal with futile care [17], overtreatment when there is no expected benefit for the patient [13], poor communication with patients and their families and with co-workers [21]. In mental health care, RNs experienced moral distress when they had to manage care for people suffering greatly and needing seclusion or coercion but where resources were lacking [11]. Physicians have described enduring moral distress when faced with too long waiting lists [22], crucial end-of life decisions in intensive care, and in the tension between ensuring a good death and saving life [23]. Hospital social workers experienced moral distress when patients were not provided with an adequate service. [24].

Moral distress is associated with organizations that have hierarchical structures, poor collaboration [18] and poor communication within teams [20].

Lack of competence in the healthcare team has also been considered an issue to moral distress [13]. Bullying, lack of inclusion [16, 25] and struggling to share the troubled emotions associated with acting against one’s moral values have been described as giving rise to bad feelings and moral distress [26]. A positive ethical climate, however, [12, 27] and professional independence at work [28] have been shown to reduce the frequency of morally distressing situations [27, 28]. With reference to gender differences, female professionals have reported greater moral distress than males [29]. Colville et al. [30] also found that more female than male professionals intended to leave their positions.

Moral distress has been shown to have a personal impact on healthcare professionals [18]. The emotions related to situations contributing to moral distress have been described as feelings of being morally conflicted, torn, uncertain, indecisive and powerless [3, 10, 26]. Strong feelings of frustration, anger, fear, and even overwhelming exhaustion have been reported [16]. The sense of being caught between personal ideals and reality and thus failing the patients caused feelings of guilt and inadequacy [11]. Healthcare professionals may not realize that the situations they encounter in their clinical work can be morally distressing or ethically challenging. Interventions with organized ethics consultations may help healthcare professionals to identify the root causes of moral distress and bring clarity to a situation [31].

The Moral Distress Scale (MDS) was developed by Corley et al. [32] in order to measure the experience of moral distress experienced by RNs in intensive care. The questionnaire was shortened from 38 items [32] to 21 items by Hamric, Blackhall [7] who then further revised it to produce the Moral Distress Scale- Revised (MDS-R) [33]. This version was based on the original version of MDS but was developed to include extended root factors in moral distress and to be appropriate for use with healthcare professionals outside intensive care. To make the instrument applicable to a variety of professions, six parallel versions of MDS-R were constructed [33]. Several studies, worldwide have used the MDS-R to measure the experience of moral distress among healthcare professionals [14, 34–37]. The MDS-R was subsequently translated and culturally adapted to a Swedish pediatric context [38]. In order to further capture the underlying reasons for moral distress, the MDS-R was further developed by Epstein et al. [39] into the Measure of Moral Distress for Healthcare Professionals (MMD-HP). This instrument is designed to capture the experience of moral distress among healthcare professionals from various professions, at the individual patient level, the system level and the team level. MMD-HP can be used as a tool in interventions to identify the root causes of moral distress related to the individual professional, the team and the unit [39]. Sweden lacks a valid and reliable instrument with which to measure healthcare professionals’ perceived moral distress in relation to patient level, system level, and team level in adult health care.

## Methods

The aim of this project was therefore to translate and make a cultural adaption of the MMD -HP to the Swedish context.

The MMD-HP was developed by Epstein et al. [39] and has a factor structure at a (1) system level, (2) patient level, (3) team level related to colleagues and (4) team level related to patients. The MMD-HP comprises 27 items in the form of statements concerning ethically difficult situations or dilemmas. The items are rated according to frequency and intensity using a Likert scale from 0 (never) to 4 (very frequent) and 0 (non) to 4 (very distressing). The final questions in the MMD-HP concern whether a person has either left or intends to leave their position because of moral distress [39].

The translation procedure followed the WHO (2020) guidelines [40]. Firstly, the designer of the MMD-HP questionnaire, Dr Hamric was contacted and permission was granted for the translation. Later, after contact with Dr Epstein, the instrument was received with recommendations about how to use the MMD-HP version. The translation process was carried out in four steps, namely, forward translation, expert panel scrutiny and back translation, pre-testing and cognitive interviewing and final version. Cultural adaptation took place throughout the working process.

### Forward translation

The original version of MMD-HP was translated into two versions by two native Swedish healthcare professionals with extensive knowledge and experience of the English language. A nurse assistant working in elderly care and a physician in cardiac and emergency care independently translated the questionnaire. The authors (MB, CFG) reviewed this forward translation of the MMD-HP and merged the two versions.

### Back translation

Differences in choice of words in every item were discussed and MB and CFG decided on the final formulations. The instrument was back translated into English by two native English translators with extensive knowledge of Swedish. The back translators worked independently and without any knowledge of the pre-translated version of the questionnaire. The authors reviewed the back-translated versions. Choices of words were analyzed and compared with the pre-translated version regarding their meaning and content. When the back-translated items contained various choices of words with the same meaning, MB and CF discussed alternative wordings used in the initial translation of the questionnaire and selected those that most closely reflected the pre-translated version.

### Expert panel

Cognitive interviews with researchers and members (n = 7) of the research group Research in Future Ethical Care Challenges (RiFeCC) were used for content validation of the back-translated version. The RiFeCC members reviewed the back-translated version in relation to the original instrument. Discussions were carried out individually (n = 2) or in groups (n = 5). Every item was discussed in relation to sentence structure, comprehensibility, clarity of definition, relevance and sensitivity, and whether or not emotions were evoked [cf. 41].

### Pretesting and cognitive interviews

To achieve face validity, content validity and respondent satisfaction, cognitive face-to-face interviews were performed with healthcare professionals from various professions and clinical contexts (n = 10) in Swedish health care. The professionals were a physiotherapist [PT] (n = 1), occupational therapist [OT] (n = 1), registered nurses [RNs] (n = 5), enrolled nurses [ENs] (n = 2) and a physician (n = 1)**;** eight female and two male professionals aged from 31 to 72 years and with 7–47 years professional experience. The interviews were conducted individually by the authors [MB, CFG], in a separate room (n = 2) or in digital meeting rooms (n = 8), using the think aloud method [cf. 41]. The participants received the questionnaire before the interview. They were asked to reflect on every item, focusing on comprehensibility, clarity of definition, relevance, sensitivity, whether or not emotions were evoked and also to make suggestions for alternative wordings. The participants read every item, voiced their concerns, and responded to statements and suggestions for improvements. At the same time the authors asked probing questions when they needed clarification [cf. 41]. The interviews were audio- (n = 2)] or audio- and video-recorded (n = 8), with the agreement of the participants, and were transcribed item by item by the authors.

## Results

The consistent ambition throughout the translation process and cultural adaptation of the MMD-HP questionnaire was to keep as close as possible to the formulation and concept of the original version. Inevitably parts of some items had to be adjusted or removed to make them relevant and comprehensible in a Swedish context. The cognitive interviews, expert panel and researchers’ discussions focused on two aspects, relevance and comprehensibility/clarity.

### Relevance

In general, the MMD-HP questionnaire was met with recognition among the interviewees. The statements were reflected over and related to experiences, conditions and situations from clinical practice. Examples of statements which forms the basis of the cultural adaption are presented in the text below and in Table 1.

**Table 1:** Examples of statements which forms the basis of the cultural adaption

Item no	English original	Comments from interviewees	Swedish cultural adaption
4	Be unable to provide optimal care due to pressures from administrators or insurers to reduce costs	‘Insurer companies? When am I pressured by that? How can an administrator have an influence on me? It works in the U.S.A or England but not here. It is not on the map that this could happen’ RN (2)	Hindrad att ge optimal vård på grund av påtryckning från ledning för att minska kostnader
6	Be pressured to avoid taking action when I learn that a physician, nurse, or other team colleague has made a medical error and does not report it	‘Why using physician and nurse when it is interprofessional [work]. It becomes an indication that these professions are more important. Team member is better’ OT (10)’ ‘Team is the form of work. One assumes that everyone is working in a team-oriented way (RN 3)’	Känna press att inte agera när jag får veta att en kollega eller annan teammedlem har gjort ett medicinskt misstag som inte rapporterats
8	Participate in care that causes unnecessary suffering or does not adequately relieve pain or symptoms	’Pain is a symptom. Add [to pain] or other symptoms’ RN (7)	Delta i vård som orsakar onödigt lidande eller som inte tillräckligt lindrar smärta eller andra symtom
12	Participate in care that I do not agree with, but do so because of fears of litigation	‘Litigation is strange. Fear is better OT (10) Fear of being reported if I do not’ PT (9)	Delta i vård som jag inte håller med om, men gör det på grund av rädsla för att bli anmäld

A nurse working in oncology care responded with recognition to item no 17: Experience compromised patient care due to lack of resources/equipment/bed capacity.

Yes! Because we may have plenty of beds but no staff, plenty of masks but no one who can do the work (RN 3)

An enrolled nurse working in intensive care agreed with item no 19: To have excessive documentation requirements that compromise patient care.

Excessive documentation is a good designation, because it covers several different systems which in the long run means double documentation. It becomes uncertain just for that reason (EN 1)

Regarding relevance, the main aspects expressed in the cognitive interviews concerned statements that were felt to be irrelevant or difficult to put into a Swedish context.

Moral distress arises when moral integrity is threatened. The condition is burdensome, evoking feelings of stress and anguish. There is no actual everyday word in the Swedish language for distress, comprising ethics, stress and anguish. Moral stress was found to be a useful term for conveying a comprehensible concept in everyday language.

The cognitive interviews revealed a culture clash in connection with the statement: Be unable to provide optimal care due to pressures from administrators or insurers to reduce costs (item no 4).

Insurer companies? When am I pressured by that? How can an administrator have an influence on me? It works in the U.S.A or England but not here. It is not on the map that this could happen (RN 2)

In Sweden healthcare is a general right, hence health insurance is funded by society. It means that neither insurers nor administrators have the power to exert pressure in order to reduce costs. Therefore, a suggestion from the interviews, to make the version more relevant to Sweden, was to change “pressure from insurers or administrators” to “pressure from management”.

Also, in the cognitive interviews the following statement: Be pressured to avoid taking action when I learn that a physician, nurse, or other team colleague has made a medical error and does not report it (item no 6) was viewed as extraordinary. In Sweden the healthcare team involves several different professions. To clarify the focus of the statement “physician, nurse” was changed to “colleague”.

Why using physician and nurse when it is interprofessional [work]. It becomes an indication that these professions are more important. Team member is better (OT 10)

Team is the form of work. One assumes that everyone is working in a team-oriented way (RN 3)

Concerning item no 8: Participate in care that causes unnecessary suffering or does not adequately relieve pain or symptoms; the cognitive interviews revealed that pain is seen as a symptom. Hence in the Swedish version the words “pain or other symptoms” are added.

Pain is a symptom. Add [to pain] or other symotoms (RN 7)

Item no 12 reads as follows: Participate in care that I do not agree with, but do so because of fears of litigation. In the Swedish context, fear of being reported is more likely than fear of litigation, thus “being reported “was substituted for litigation.

Litigation is strange. Fear is better (OT 10)

Fear of being reported if I do not’ (PT 9)

### Comprehensibility/clarity

The main aspects related to comprehensibility/clarity expressed in the cognitive interviews concerned sentences, wordings and the accessibility of their meaning. Statements that lacked flow, were difficult to voice or which needed to be repeated, inspired suggestions for improvements.

Words such as “being under pressure” (items 3, 6 and 15) were changed from “being pressured” to “feeling press to” (*Känna press*). The phrase, “being required”, implies a demand, as in “being ordered to” in Swedish. “Having to” (*måste*) (items 7, 22) or “expected to” (*förväntas*) are more common in everyday speech (item 13) and were therefore used instead.

Regarding item 4: Be unable to provide optimal care due to pressures from administrators or insurers to reduce costs; hindered from (*hindrad*) was chosen as a more appropriate word since the statement concerns inability to act due to external factors. For the sake of consistency, concerning the concept of patient–person, person was changed to patient throughout the questionnaire (item 5). In order to make the following statement more comprehensible: Witness low quality of patient care due to poor team communication; (item 14) poor communication was replaced by “deficient” (*bristande*) communication.

Item 15: Feel pressured to ignore situations in which patients have not been given adequate information to ensure informed consent; (item 15) contains a repetition. The interviewees had to talk and think about the idea of the sentence. “informed” was removed, resulting in the statement becoming clearer.

Lack of information automatically leads to lack of informed consent (RN 2)

The use of compromised/compromising/compromise (Swedish *äventyra kompromissa*) (item 17, 18, 19) was found to blur the meaning of the statements. Replacing those words with “deteriorate” (*försämras*/ *försämrad*), clarified the content of the Swedish version of the statement. Further on in item 19: Have excessive documentation requirements that compromise patient care; “have” is replaced by experience (*upplever*). The expert group and cognitive interviews found that item 24: Be required to care for patients who have unclear or inconsistent treatment plans or who lack goals of care; lacked clarity. As the item contains two statements they felt it was not clear what the content was about or how they should respond.

*‘The last part, who lack goals of care feels fuzzy (PT 9) ’* In the Swedish version the last part of sentence “or who lack goals of care” was removed.

## Discussion and methodological considerations

The aim of this project was to translate and make a culturally adapted version of the MMD-HP from English into the language and culture of Sweden. The WHO (2020) [40] guidelines were followed throughout the translation process. Several different methods for cultural adaptation and language translation of questionnaires have been described. According to Epstein et al. [42] any of the validated methods can be useful if the process is rigorous and equivalence with the original version is maintained. The ambition throughout this translation and cultural adaptation process of the MMD-HP was to keep items as close as possible to the original version. In cognitive think aloud interviews, healthcare professionals from various professions and contexts discussed the comprehension and relevance of the translated version, expressed recognition of experiences from clinical work and suggested improvements when needed. Hence, some of the items have been reformulated in order to adapt to and be congruent with the Swedish language. Willis, Artino Jr [41] suggests that think aloud interviews are a legitimate tool for validating the content of a questionnaire as it is possible to follow the thought process of the interviewee. According to Epstein et al. [42] cognitive interviews are an important step in the translation process, disclosing misunderstandings, misinterpretations and the level of comprehensibility of the questions.

The cognitive interviews revealed that some statements in the MMD-HP required cultural adaptation to make them relevant in a Swedish context. Since the idea of moral distress is not a natural definition in everyday Swedish, moral stress was found to be a more useful term. Although the two concepts have different meanings in English, it was considered essential to make the basic concept understandable for the responders. According to Lützen [5] Moral distress as well as moral stress are psychological reactions that comes out from stress related moral problems. The movement from moral distress to moral stress is in line with an earlier translation and cultural adaptation of MDS-R for use in a Swedish pediatric context [cf. 38]. The statement in item 4 concerning the power of administrators and insurers to impact on the possibilities for providing good patient care was seen as representing a culture clash, because of the organization of Swedish health care. Hence “pressure from management” was found to describe a more realistic situation in the Swedish context. This change is also found in the pediatric version of MDS-R by Sandeberg et al. [38]. Despite language and cultural adaptation, the final version of the MMD-HP in Swedish is close and equivalent to the original version in terms of concept, items and meaning of items [cf. 42].

The MMD-HP questionnaire was developed to measure the experience of moral distress among various healthcare professionals from different healthcare contexts, including extended underlying factors on a team level. Hamric [4] emphasizes that the experience of moral distress concerns all professions represented in the healthcare team. Healthcare professionals experience moral distress in various ways and for different reasons and it is, therefore, essential for the professionals to communicate and share experiences [4]. In an intervention study measuring the experience of moral distress before and after reflective debriefing among nurses the MDS-R was found to be valid for assessing the root causes of moral distress [43]. MMD-HP, involving additional root causes on the team level [39], has been found useful for measuring moral distress among Japanese physicians and nurses [44]. Previous qualitative studies revealed that inter-professional ethical communication in groups promoted broadened perspectives, trust and improved understanding among healthcare professionals from different professions and contexts [45, 46]. MMD-HP was translated and adapted to a Swedish context in order to examine how moral distress is affected by an intervention comprising ethics communication in groups among healthcare professionals from various professions and care contexts. The Swedish version of MMD-HP can be useful in measuring moral distress before and after such an intervention in a Swedish healthcare context.

## Conclusion

The Measure of Moral Distress for Healthcare Professionals, MMD-HP, was translated from English and adapted to the Swedish language and culture. Despite some cultural adaptations, the final Swedish version was equivalent to the original version. The Swedish version of MMD-HP can be useful in measuring moral distress among healthcare professionals in a Swedish healthcare context. In order to determine content validity of the instrument future research with psychometric tests are required.

## Data Availability

The audio, audio–video recorded interviews and transcripts in this study are not available in published form by the respect for the participants’ confidentiality. However, with reasonable reasons data is available by the corresponding author.

## Abbreviations

MMD-HP: Measure of Moral Distress for Healthcare Professionals
MDS: Moral Distress Scale
MDS-R: Moral Distress Scale- Revised
